# Effectiveness of Mindfulness-Based Stress Reduction Intervention for Health-Related Quality of Life in Drug-Dependent Males

**DOI:** 10.5812/ircmj.12608

**Published:** 2014-09-05

**Authors:** Navid Reza Hosseinzadeh Asl, Fattaneh Hosseinalipour

**Affiliations:** 1 Institute of Social Sciences, Hacettepe University, Ankara, Turkey; 2 Institute of Educational Sciences, Gazi University, Ankara, Turkey

**Keywords:** Drug Addiction, Mindfulness-Based Stress Reduction, Health-Related Quality of Life

## Abstract

**Background:**

Nowadays drug dependence has become one of the main concerns worldwide. Indeed, drug dependence and abuse have become personal, social and, health problems that intensively threat human resources.

**Objectives:**

This study aimed to examine the effect of mindfulness-based stress reduction (MBSR) on health-related quality of life (HRQOL) in drug-dependent males.

**Patients and Methods:**

An experimental research with pretest/posttest design and a control group was conducted on 49 drug-dependent males. The selected individuals were randomly allocated to the study groups (24 to the experimental and 25 to the control groups). Afterwards, the Short Form Health Survey for HRQOL (SF-36) questionnaire was completed by participants. Subsequently, the experiment group experienced eight 90-minute sessions of training in MBSR. At the end of the training, the subjects were evaluated once again by SF-36. The independent-samples t test and analysis of covariance (ANCOVA) were used to analyses data.

**Results:**

The results suggested that the mean differences between groups regarding scales of role emotional, vitality, mental health, social functioning, and bodily pain were statistically significant (P < 0.05).

**Conclusions:**

Using MBSR is recommended for improving HRQOL in drug-dependent males who are undergoing treatment for their drug dependence in addiction treatment center; however, more studies concerning different drugs as well as drug dependence in women must be performed.

## 1. Background

Nowadays drug dependence has become one of the main public health concerns worldwide (1). Indeed, drug dependence and abuse have been turned to personal, social, and health problems that intensively threat human resources (2). Drug abuse has a negative influence on many parts of life such as physical, psychological, social, economic, and familial aspects. Results of a research that evaluated the role of the social and clinical variables in quality of life (QOL) of addicts showed that personality disorders, interpersonal conflicts within a family and with spouse, and the need for treatment of physical and mental disorders were significantly associated with lower QOL (3).

Findings of a study by Tracy et al. suggested that the various domains of QOL of the addicts were less than that of normal subjects. The history of injuries/traumas could significantly predict the level of QOL in mental and physical domains (4). QOL is defined in different ways such as health-related QOL (HRQOL). The term HRQOL is used in measuring the influence of various irregularities, infirmity, and long-term as well as short-term illnesses in different populations. In fact, HRQOL is used for evaluating daily life effects on health (5). The findings of the research by Khajedaluee et al. demonstrated that drug abuse led to reduced HRQOL in all domains in comparison with control group and suggested that different aspects of HRQOL in addicted people were impaired (6).

Mindfulness-based stress reduction (MBSR), which is aimed at reduction of psychologic symptoms of distress and enhancement of QOL, is increasingly applied in various settings in both mental and somatic healthcare (7, 8). These interventions are aimed at cultivating an open-minded and nonjudgmental awareness of whatever is happening at each successive moment of perception. The objects of perception, which is direct and prereflexive, include the whole range of possible phenomena from internal psychologic states and processes (thoughts, feelings, images, etc.) and proprioceptive information from the body to the external stimuli entering the senses. Phenomena are approached in an open, nonjudgmental, and accepting way. It is “the clear and single-minded awareness of what actually happens to us and in us at the successive moments of perception” (9). Beneficial effects have been reported in diverse samples of patients and general population regarding quality of life, which state that MBSR can improve HRQOL (10, 11).

## 2. Objectives

There were few studies regarding the effects of MBSR on drug-dependent individuals’ HRQOL; therefore, the present study aimed to determine the MBSR influence on HRQOL of drug-dependent males in order to facilitate the treatment and to eliminate the existed barriers on their recovery process. In other words, the following hypothesis was examined: MBSR is effective in increasing of HRQOL in drug-dependent males.

## 3. Patients and Methods

### 3.1. Subjects

This experimental study used a pretest-posttest controlled approach (12). The statistical population of this study was comprised of drug-dependent males in Tabriz, Iran. After obtaining approval of the local ethic committee for clinical works and studies, study samples were recruited from an addiction treatment clinic. The participants were 53 drug-dependent males who regularly attended the addiction treatment center. All subjects were opium or heroin dependent and were under methadone maintenance treatment during the study. The mean age of the participants was 36.8 years (range, 19-46). Four subjects did not continue to process and therefore, the remaining 49 participants were randomly allocated to two groups: experiment, 24 patients; and control, 25 patients. Subsequently, all participants answered the Short Form of Health Survey for HRQOL (SF-36). Then the MBSR was performed for the experiment group while control group received no therapy. At the end of the eighth sessions, the participants of both groups answered SF-36 for the second time.

### 3.2. Questionnaire

The SF-36 has eight scales including physical function, role physical, role emotional, vitality, mental health, social functioning, bodily pain, and general health. The standard Farsi version of SF-36 with the reported coefficient ranging from 65% to 90% for the eight scales, which shows good inner persistency of these scales, was used. Other measuring such as getting the validity was considered, which verified suitability of the tool in this population (13).

### 3.3. Brief Manual for the Use of Mindfulness-Based Cognitive Therapy

The training sessions were arranged according to the described MBSR program by Kabat-Zinn (7). The treatment period included eight sessions of group training, which were held as 90-minute group sessions weekly. During these sessions, experimental subjects were trained to gain an ability to keep themselves away from their thoughts and feelings and instead, focus on the changes occurring in their body and mind. Clients were encouraged to adopt a new way of being and relating to their thoughts and feelings, while placing little emphasis on altering or challenging specific cognitions. In some sessions, poems with mindfulness content were used for clients to help them in concentration. Poems by Iranian poets (Sohrab Sepehri, Nader Naderpour, and Shafiee Kadkani) were also used. In addition, audio tapes and books concerning deep relaxation were used to guide clients in performing mindfulness exercises at home. It should be noted that once the study was terminated, for ethical reasons, the control group also received the MBSR.

### 3.4. Data Analysis

The scores of SF-36 scales obtained at pretest and posttest were analyzed using the independent-samples t test and Analysis of Covariance (ANCOVA) by SPSS 22.

## 4. Results

The independent-samples t-test was conducted to compare experimental and control groups at the pretests for each SF-36 scale. There was no statistically significant difference between the mean scores of the experimental and control groups at any SF-36 scale before starting the study (P > 0.05), implying that the two groups were comparable in terms of HRQOL before the study. ANCOVA was performed to compare experimental and control groups at each SF-36 scale at the end of study (Table 1).

**Figure 1.**
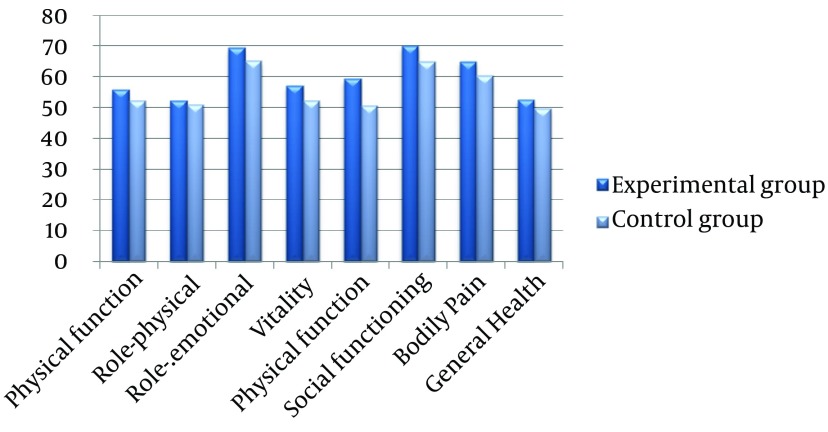
Posttests Mean Scores for SF-36 Scales

The posttest mean SF-36 scores of all scales for the experimental group were higher in comparison with the controls (Figure 1). However, the mean scores for scales of role emotional, vitality, mental health, social functioning, and bodily pain were significantly different between study groups (P < 0.05). Thus, the research hypothesis that “MBSR is effective in increasing HRQOL in drug-dependent males” was confirmed for these scales.

**Table 1.** Comparisons Between Mean Scores of Groups at Posttest ^a,b^

SF-36	No.	Mean ± SD	df	Mean Square	F	P value	ɳ^2^	Observed Power
**Physical Function**			1	60.17	3.28	0.077	0.066	0.426
E	24	55.83** ± **9.93						
C	25	52.36** ± **9.00						
**Role Physical**			1	59.423	1.830	0.183	0.038	0.263
E	24	52.25** ± **6.96						
C	25	50.84** ± **8.99						
**Role Emotional**			1	172.78	6.21	0.016	0.119	0.685
E	24	69.46** ± **5.61						
C	25	65.20** ± **6.84						
**Vitality**			1	495.42	21.97	0.000	0.323	0.996
E	24	57.25** ± **8.25						
C	25	52.32** ± **7.65						
**Mental Health**			1	769.84	11.11	0.002	0.195	0.904
E	24	59.38** ± **9.16						
C	25	50.60** ± **8.75						
**Social Functioning**			1	248.2	5.63	0.022	0.109	0.642
E	24	70.04** ± **6.83						
C	25	64.88** ± **9.84						
**Bodily Pain**			1	241.97	5.71	0.021	0.110	0.648
E	24	64.96** ± **5.87						
C	25	60.44** ± **6.94						
**General Health**			1	61.12	1.99	0.165	0.042	0.282
E	24	52.75** ± **6.75						
C	25	49.68** ± **7.19						

^a^ Abbreviations: E; experimental group, C; control group, SD; standard deviation; and df, degree of freedom.

^b^Significance level is < 0.05. ɳ^2^ stands for squared partial eta.

## 5. Discussion

According to our results, MBSR was a useful method for enhancing the HRQOL in drug-dependent males. This finding was in agreement with those of previous studies that reported the effectiveness of MBSR on HRQOL; however, they concerned those other than drug-dependent males. In a study on outpatients with breast and prostate cancer, Carlson et al. reported the significant effect of MBSR on overall QOL, symptoms of stress, and sleep quality improvements (10). In a research by Nyklicek and Kuijpers, the experiment group showed significantly stronger reductions of perceived stress and vital exhaustion as well as stronger improvement of QOL and mindfulness in comparison to controls (11). In a meta-analysis study, Grossman et al. reported that MBSR might help a broad range of individuals to cope with their clinical and nonclinical problems (14).

Findings of the present study confirmed the association of cultivating a more mindful way of being with less emotional distress and a more positive state of mind. Increased awareness of thoughts and emotions, acceptance, and compassion appear to promote optimal HRQOL. The awareness of drug-dependent individuals of their negative and positive emotions appears to play an important role in their recovery and adjustment to their states. However, additional well-designed studies using larger samples of drug-dependent individuals and active control groups are needed to replicate and verify the mental health benefits of mindfulness meditation training. The findings of this study supported a relationship between cultivating a more mindful way of being and a tendency to experience better HRQOL. Considering a relationship between poor HRQOL and addiction (6), the development of short-term efficient therapeutic techniques such as MBSR for increasing HRQOL is crucial. The generalization of the results of the present study was limited by the small sample; hence, future research should focus on larger and more heterogeneous samples of individuals with substance use disorders.
